# The psychological impact of the COVID-19 pandemic on secondary school teachers in Sfax, Tunisia : anxiety disorders

**DOI:** 10.1192/j.eurpsy.2022.856

**Published:** 2022-09-01

**Authors:** N. Regaieg, L. Zouari, Y. Mejdoub, S. Omri, I. Gassara, R. Feki, N. Smaoui, M. Maalej, J. Ben Thabet, N. Charfi, M. Maalej

**Affiliations:** 1 Hedi Chaker University Hospital, Psychiatry C, Sfax, Tunisia; 2 Hedi Chaker University Hospital, Epidemiology, Sfax, Tunisia

**Keywords:** Anxiety, Covid-19, secondary school teachers

## Abstract

**Introduction:**

During the COVID-19 pandemic time, teachers have to adapt to many changes that could potentially make them more vulnerable to psychological distress.

**Objectives:**

To determine the prevalence and the factors for anxiety during the COVID-19 epidemic among the high school teachers in Sfax, Tunisia.

**Methods:**

This was a cross-sectional study, for descriptive and analytical purposes, conducted on google drive in May 2021.We involved 97 junior and secondary school teachers from Sfax, Tunisia, practicing in public high schools. Anxiety was assessed by using Generalized Anxiety Disorder tool (GAD-7).

**Results:**

The average age of the participants was 44.23 years old with a sex-ratio (M/F) of 0.32. Since the advent of COVID-19, 54.2% had presented sleep disturbances while 10.4% had suicidal thoughts. Among all participants, 77.8% were afraid of the virus transmission within the classroom or the school and 81.3% reported regular mask wear at work. The median score on the GAD-7 was 6.5 (Q1=3, Q3=11). Scores’ distribution indicated that 68% of the participants had no to mild symptoms of anxiety while 32% had moderate to severe anxiety. Furthermore, the presence of anxiety was associated to the female gender (p=0.01), sleep disturbances (p<0.001), suicidal thoughts (p=0.006), and to the conviction that wearing masks blocks the transmission of voice, information or emotions between teacher and student (p=0.025).

**Conclusions:**

During the COVID-19 outbreak, high school teachers in Sfax, Tunisia have high levels of anxiety.This can lead to a decreased effectiveness of their complex work.

**Disclosure:**

No significant relationships.

